# *Euonymus alatus*: A Review on Its Phytochemistry and Antidiabetic Activity

**DOI:** 10.1155/2016/9425714

**Published:** 2016-08-25

**Authors:** Xifeng Zhai, George Binh Lenon, Charlie C. L. Xue, Chun-Guang Li

**Affiliations:** ^1^Traditional & Complementary Medicine Program, School of Health Sciences, RMIT University, Bundoora, VIC 3083, Australia; ^2^School of Pharmaceutical Sciences, Xi'an Medical University, Xi'an 710021, China; ^3^National Institute of Complementary Medicine, Western Sydney University, Penrith, NSW 2751, Australia

## Abstract

*Euonymus alatus (E. alatus)* is a medicinal plant used in some Asian countries for treating various conditions including cancer, hyperglycemia, and diabetic complications. This review outlines the phytochemistry and bioactivities of* E. alatus* related to antidiabetic actions. More than 100 chemical constituents have been isolated and identified from* E. alatus*, including flavonoids, terpenoids, steroids, lignans, cardenolides, phenolic acids, and alkaloids. Studies* in vitro* and* in vivo* have demonstrated the hypoglycemic activity of* E. alatus* extracts and its certain constituents. The hypoglycemic activity of* E. alatus* may be related to regulation of insulin signaling and insulin sensitivity, involving PPAR*γ* and aldose reductase pathways. Further studies on* E. alatus* and its bioactive compounds may help to develop new agents for treating diabetes and diabetic complications.

## 1. Introduction 

*Euonymus alatus (E. alatus) *is a medicinal plant used traditionally in many Asian countries, including China and Korea, for treating various conditions. It has long been used in China as a Chinese Materia Medica for pain and menstrual disorders. The first record of its clinical use in China was documented in* Shen Nong Ben Cao Jing* (The Classic of Herbal Medicine) written between 300 BC and 200 AD.* Ben Cao Gang Mu* (Compendium of Materia Medica, AD1578, written by Li Shizhen) later recorded its applications for vaginal bleeding, abdominal distention, and detoxification, and* Ben Cao Jing Ji Zhu* (Collective Notes to Canon of Materia Medica) recorded its use for abdominal pain, killing worms, and eliminating skin swelling caused by various reasons [1]. The interest in* E. alatus* has been increased recently largely due to the research on its bioactivities against cancer and diabetes. Recent studies have demonstrated a wide range of bioactivities of* E. alatus*, including hypoglycemic, antihypertensive [2], antitumor [3, 4], sedative [2], and regulation of blood lipid [5, 6] and immune functions [7]. There is also clinical evidence for its efficacy against hyperglycemia [8], chronic nephropathy [9], rheumatoid arthritis [10], cor pulmonale [11], bronchial asthma [12], anaphylactic disease [13, 14], urinary tract infection, and prostate diseases [15]. This short review outlines the phytochemistry of* E. alatus* and its pharmacology related to antidiabetic actions.

## 2. Phytochemistry

More than 128 chemical constituents have been isolated and identified from* E. alatus*. The main chemical classes include flavonoids, terpenoids, steroids, lignans, cardenolides, phenolic acids, and alkaloids.

### 2.1. Flavonoids

A total of 26 flavonoids have been isolated and identified from* E. alatus*. The main structure types include flavonoid, flavanone, and flavonol. The aglycones of flavonoid glycosides isolated from* E. alatus* include quercetin, kaempferol, naringenin, aromadendrene, and dihydroquercetin. The flavonoids are mainly distributed in the leaves and wings of* E. alatus* [16]. The structures of main flavonoids identified in* E. alatus* are listed in Tables 1 [Table tab2] [Table tab3]–4.

Other flavonoids include catechin (19) [17–21], symplocoside (20) [17], quercetin-3-galactosyl-xyloside (21) [20], catechin lactone A (22) [17], dehydrodicatechin A (23) [17–21], 3-hydroxycoumarinflavanol (24), 7,4′-dihydroxy-8-C-glucoxylisoflavone (25) [22], and 5-hydroxy-6,7-dimethoxyflavone (26) [23].

### 2.2. Steroids

Eight steroids including sterols and sterones have been isolated and identified from* E. alatus*. The main structures of the steroids are shown in Table 5. Other steroids include 24R-methyllophenol (34) and *α*-spinasterol (35) [22].

### 2.3. Terpenoids

The main terpenoids isolated from* E. alatus* include triterpenes and sesquiterpenes.

#### 2.3.1. Triterpenes

Multiple types of triterpenes were found in* E. alatus*. Most of the triterpenes in* E. alatus* belong to lupane type and oleanane type. Other types include hopane, ursane, and friedelane.  Table 6 shows the lupane type and friedelane type triterpenes isolated from* E. alatus.*

**Figure 1:**
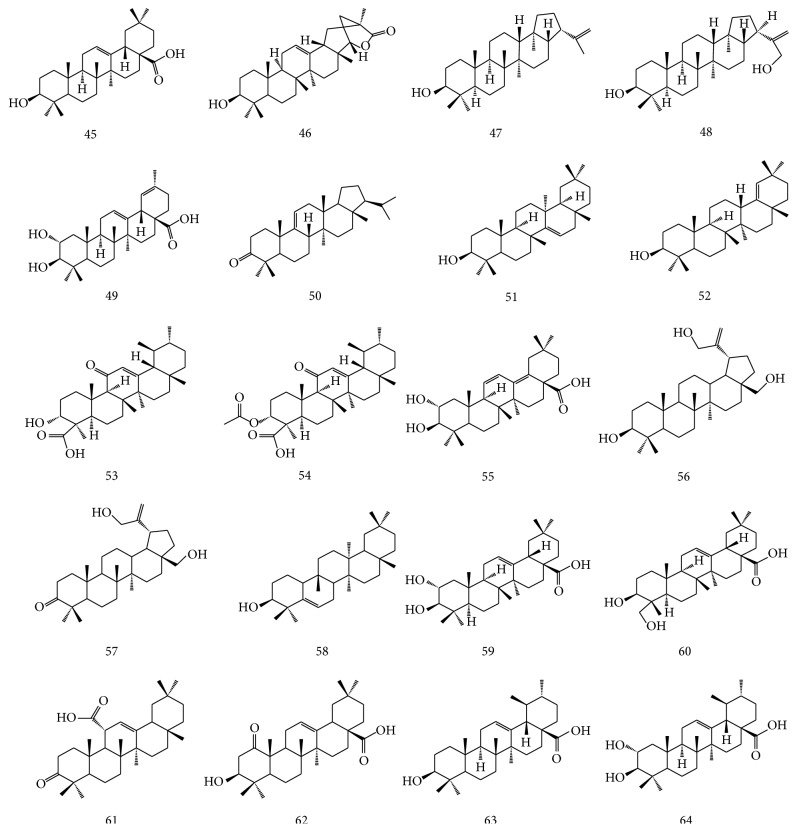
The structures of compounds 45–64 isolated from* E. alatus.*

Other triterpenes include oleanic acid (45), wilforlide A (46) [24], hop-(22)-29-en-3*β*-ol (47) [25], 3*β*-hydroxy-21*α*H-hop-22(29)-en-30-ol (48), 2*α*,3*β*-dihydroxyurs-12,19-dien-23,28-oic acid (49) [21], arborinone (50), taraxerol (51) and germanicol (52) [22], 11-keto-*β*-boswellic acid (53), acetyl 11-keto-*β*-boswellic acid (54), camaldulenic acid (55) [23], 3*β*,28,30-lup-20(29)-ene triol (56), 28,30-dihydroxy-3-oxolup-20(29)-ene (57), glut-5-en-3*β*-ol (58), maslinic acid (59), hederagenin (60), 3-oxo-11alpha-methoxyolean-12-ene (61), 3*β*-hydroxy-1-oxo-olean-12-en-28-oic acid (62), ursolic acid (63), and 2*α*-hydroxy-ursolic acid (64) [26]. The structures of compounds 45–64 are shown in Figure 1.

#### 2.3.2. Sesquiterpenes

**Figure 2:**
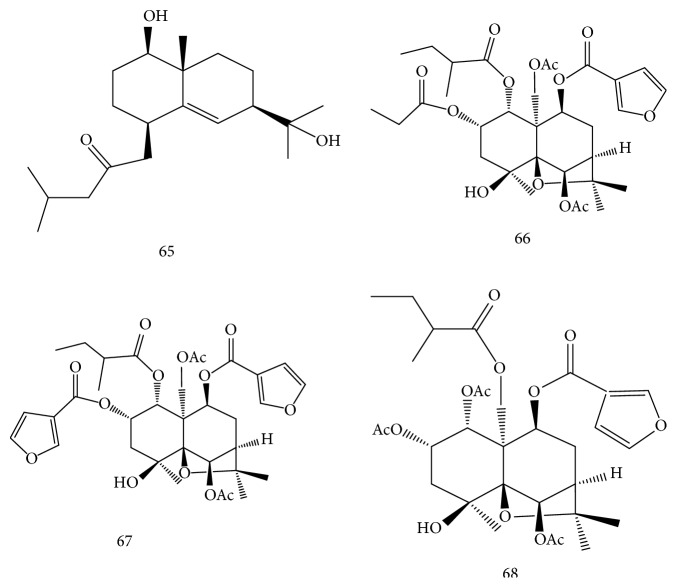
Structures of sesquiterpenes (compounds 65–68) isolated from* E. alatus.*

Two new sesquiterpenes (65, 66) and two known ones were isolated from 95% ethanol extract of the stems of* E. alatus*. The known ones were identified as 6*α*,12-diacetoxy-2b,9*α*-di(b-furancarbonyloxy)-4*α*-hydroxyl-1*β*-(2-methylbutanoyloxy)-*β*-dihydroagarofuran (67), 1*α*,2*α*,6*β*-triacetoxy-4*β*-hydroxy-9*β*-(*β*-) furancarboxy-15-[(amethyl) butyroyloxy]-*β*-dihydroagarofuran (68) [27]. The structures of sesquiterpenes isolated from* E. alatus* are shown in Figure 2.

### 2.4. Alkaloids

**Figure 3:**
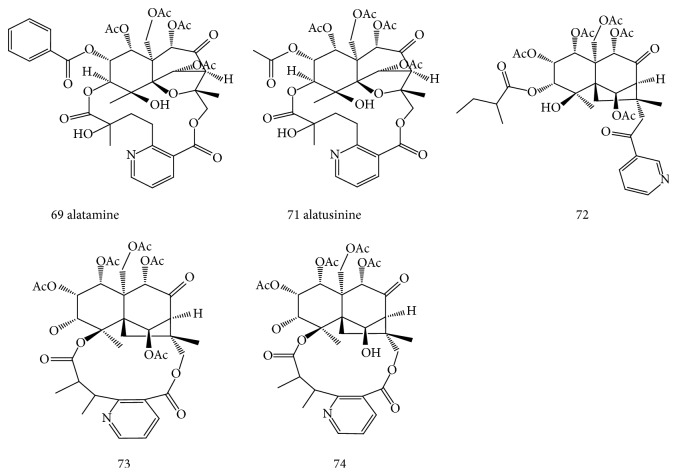
Structures of alkaloids (compounds 69 and 71–74) isolated from* E. alatus.*

Five alkaloids have been isolated from* E. alatus* and identified as alatamine (69), alatusamine (70) and alatusinine (71) [28], 1*β*,2*β*,5*α*,8*β*,11-pentaacetoxy-4*α*-hydroxy-3*α*-(2-methylbutanoyl)-15-nicotinoyl-7-oxo-dihydroagarofuran (72), evonine (73), and neoevonine (74) [27]. The structures of alkaloids isolated from* E. alatus* are shown in Figure 3.

### 2.5. Cardenolides

Kitanaka et al. [3] isolated three cytotoxic cardenolides from the woods of* E. alatus* and identified them as acovenosigenin A 3-O-*α*-L-rhamnopyranoside (75), euonymoside A (76), and euonymusoside A (77).

### 2.6. Lignans

**Figure 4:**
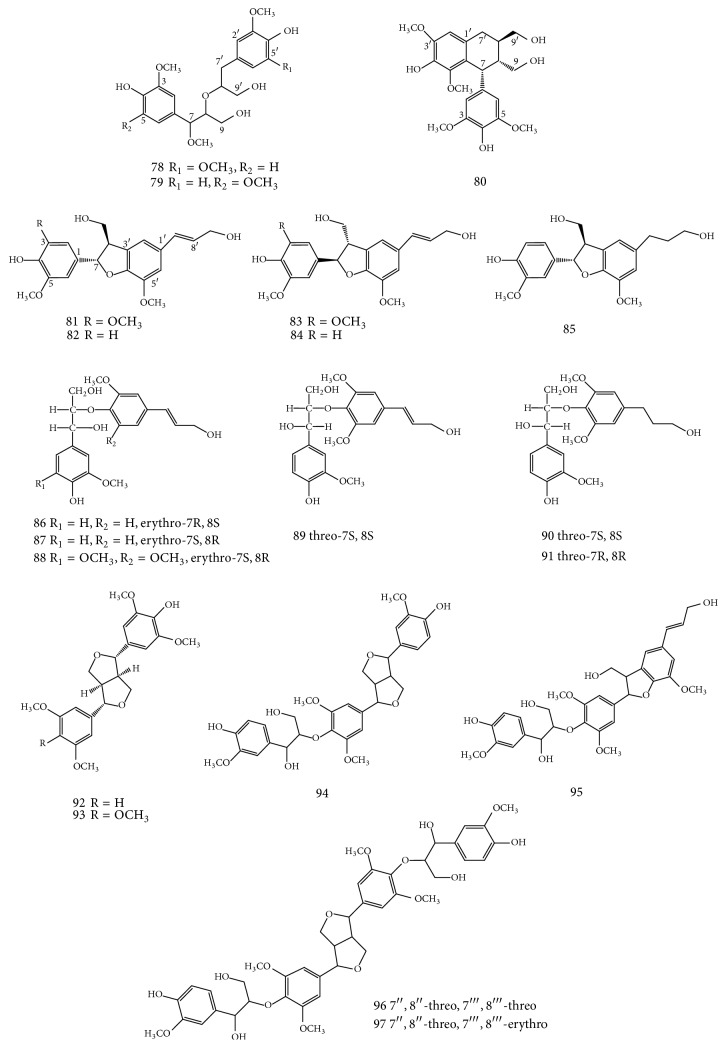
Structures of compounds 78–97 isolated from leaves and twigs of* E. alatus*, modified from [29].

Jeong et al. [29] identified three new lignans from 80% methanolic extract of* E. alatus* leaves and twigs, including (−)-threo-4,9,4′,9′-tetrahydroxy-3,7,3′,5′-tetramethoxy-8-O-8′-neolignan (78), (−)-threo-4,9,4′,9′-tetrahydroxy-3,5,7,3′-tetramethoxy-8-O-8′-neolignan (79), and (7R,8R,7′R)-(+)-lyoniresinol (80). The other known compounds identified include (+)-simulanol (81), (+)-dehydrodiconiferyl alcohol (82), (−)-simulanol (83), (−)-dehydrodiconiferyl alcohol (84), (+)-dihydrodehyrodiconiferyl alcohol (85), 7R,8S-guaiacylglycerol-8-O-4′-(coniferyl alcohol) ether (86), 7S,8R-guaiacylglycerol-8-O-4′-(coniferyl alcohol) ether (87), 7S,8R-syringylglycerol-8-O-4′-(sinapyl alcohol) ether (88), 7S,8S-guaiacylglycerol-8-O-4′-(sinapyl alcohol) ether (89), 7S,8S-4,9,9′-trihydroxy-3,3′-dimethoxy-8-O-4′-neolignan (90), 7R,8R-4,9,9′-trihydroxy-3, 3′-dimethoxy-8-O-4′-neolignan (91), (+)-syringaresinol (92), de-4′-methylyangabin (93), hedyotol C (94), threo-buddlenol B (95), hedyotisol C (96), and hedyotisol B (97). The structures of compounds 78–97 are shown in Figure 4.

### 2.7. Other Constituents

*E. alatus* also contains organic acids, esters, and aldehydes, as illustrated examples in Table 7.

In addition, 3,4-dihydroxybenzoic acid (114), p-propoxybenzoic acid (115), p-coumaric acid (116), ferulic acid (117), 1-feruloyl-*β*-D-glucoside (118), tetradecyl (E)-ferulate (119) [22], ethyl 2,4-dihydroxy-6-methylbenzoate (120), 4,4′-dimethoxy-1,1′-biphenyl (121) [23], squalene (122) [25], 1-octacosanol (123) [24], n-hexacosanoic acid (124) [18, 24], 1,30-triacontanediol (125), tetracosanoic acid (126), n-octane (127), and n-nonane (128) [21] were also isolated from* E. alatus*. In a study of essential oil from* E. alatus* by using GC-MS, 56 volatile components were identified. The main volatile components include carboxylic acid, aldehyde, ketone, terpenoid, and derivatives of oxygenated terpenoid. Among these the highest content is hexadecanoic acid (39.69%), followed by wintergreen (5.02%) [30].

## 3. Antidiabetic Activity

The effects of* E. alatus* extracts have been tested* in vivo*. In streptozotocin (STZ) treated diabetic rats, an aqueous extract of* E. alatus* reduced the body weight, the fasting plasma glucose level, and glucose tolerance. The serum levels of insulin, glucagon, cholesterol, and triglyceride were also reduced [31]. Similar results were obtained in high-fat plus low dose STZ diabetic rats, showing that* E. alatus* treated rats had lower levels of fasting blood glucose and insulin and decreased levels of blood lipids and inflammatory mediators (TNF-*α*, C-reactive protein), indicating that* E. alatus* can improve the glucose-lipid metabolism and insulin resistance in diabetic conditions [32]. Park et al. also demonstrated that an ethanol extract of* E. alatus *reduced the body weight, increased insulin sensitivity, and corrected the associated hyperinsulinemia and hyperlipidemia in high-fat diet-induced hyperglycemic and hyperlipidemic ICR mice [33].

**Figure 5:**
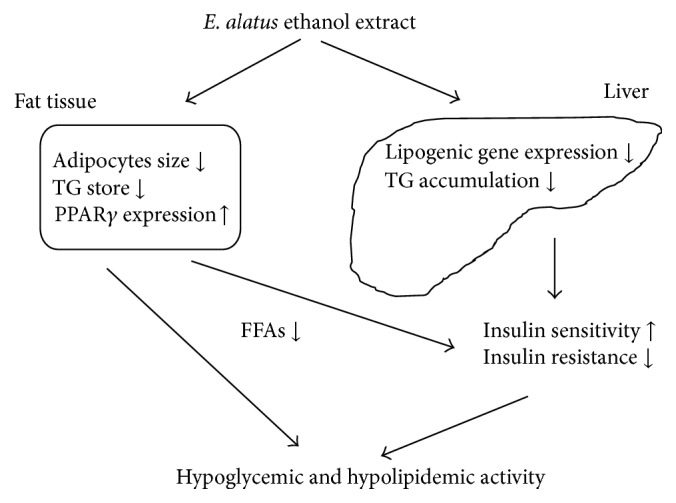
Possible mechanism of hypoglycemic and hypolipidemic actions of* E. alatus* ethanol extract, modified from [33].

The antihyperglycemic effect of* E. alatus *may involve a protection of functional islet *β* cells since* E. alatus* treated animals were shown with more positive staining of islet *β* cells than those in diabetic controls [34]. Other studies in ICR mice indicate that* E. alatus *may affect glucose and lipid homeostasis via a regulation of hepatic lipogenesis related genes (SREBPla, FAS, and GAPT) and PPAR*γ* gene expressions in periepididymal fat. The plausible mechanism of hypoglycemic and hypolipidemic actions of* E. alatus* extract is illustrated in Figure 5 [33].

In addition, a study showed that* E. alatus* protected rats from experimental diabetic nephropathy induced by uninephrectomy plus STZ treatment, with 12-week administration of* E. alatus* extract and irbesartan (positive control) decreased HbA1c and pathological changes (extracellular matrix expansion and glomerulosclerosis) in kidney and improved blood lipids profile and kidney function; the effect was associated with a downregulation of transform growth factor *β*_1_ expression [35]. In addition,* E. alatus* was shown to inhibit polyol pathway, which is known to be associated with chronic diabetic complications such as neuropathy, nephropathy, and retinopathy [36].

Fang et al. studied the antidiabetic effects of different fractions of* E. alatus* extracts (including petroleum ether, diethyl ether, ethyl acetate, n-butanol, and water fraction) in alloxan-induced diabetic mice and high-fat diet diabetic mice and found that ethyl acetate fraction significantly reduced plasma glucose and glucose tolerance in both normal and diabetic mice [37] and also reduced total cholesterol and triglyceride contents and increased SOD activity in diabetic mice [37]. Further analysis revealed that the main components in the ethyl acetate fraction were flavonoids and phenolic acids, including quercetin and kaempferol, which were known for their antioxidant activities [37]. In another study, different extract fractions of* E. alatus*, including aqueous, diethyl ether, and ethyl acetate fractions, were tested in alloxan induced diabetic mice at a dose of 10 g/kg and it was found that the aqueous extract was the most active in decreasing blood glucose and lipid levels and improved glucose tolerance [6]. Thus,* E. alatus* may contain multiple active antidiabetic constituents. Similarly, a study on the hypoglycemic fractions of six fractions of* E. alatus* extracts (including petroleum ether, ethyl acetate, n-butanol, water, residue, and rectified polysaccharide) in diabetic rats found that the fractions of petroleum ether, water, and ethyl acetate had significant antidiabetic effects. Fractions of n-butanol and rectified polysaccharide reduced blood creatinine, and other fractions reduced urea level. The residue fraction decreased the low-density lipoprotein (LDL) and cholesterol contents. The body weight was increased by the treatment with all fractions except rectified polysaccharide. These results indicate that different active compounds in these fractions may be responsible for the observed effects of* E. alatus*, including antidiabetic, antihyperlipidemic, kidney function improvement, blood viscosity decrease, and body weight affecting [38], and the active antidiabetic compounds are likely to be from the petroleum ether, water, and ethyl acetate fractions. In another study, an ethyl acetate extract of* E. alatus* was shown with hypoglycemic effect, and four compounds were isolated from this fraction and identified as p-hydroxybenzoic acid (EA-1), protocatechuic acid (EA-2), 4-hydroxy-3-methoxybenzoic acid (EA-3), and 3, 5-dimethoxy-4-hydroxybenzoic acid (EA-4) [8]. Others reported identification of six compounds with hyperglycemic activity from the 90% ethanol extracts of* E. alatus*, including aromadendrin, epifriedelanol, protocatechuic acid, *β*-sitosterol, quercetin, and rutin [39]. The active components in protecting experimental diabetic nephropathy as mentioned above have also been suggested to be concentrated in ethyl acetate and n-butanol fractions [36, 40], though the nature of these compounds is still not identified.

Jeong et al. (2015) studied the inhibitory effects of 23 compounds isolated from* E. alatus* on protein tyrosine phosphatases 1B (PTP1B) and *α*-glucosidase activities and found that lupenone, lupeol, taraxerol,* p*-propoxybenzoic acid, 1-feruloyl-*β*-D-glucoside, and 3-hydroxycoumarinflavanol exhibited inhibitory activity against PTP1B with IC_50_ values ranging from 5.6 to 18.4 *μ*M. 24R-methyllophenol, arborinone, and* p*-propoxybenzoic acid were shown with a similar activity with IC_50_ values of 15.1, 23.6, and 14.8 *μ*M, respectively. On the other hand,* p*-propoxybenzoic acid, tetradecyl (E)-ferulate, and 3-hydroxycoumarinflavanol exhibited inhibition on *α*-glucosidase with IC_50_ values of 10.5, 9.5, and 9.1 *μ*M, respectively [22].

Studies on kaempferol and quercetin, the active constituents of* E. alatus, *demonstrated that these compounds improved insulin-stimulated glucose uptake in mature 3T3-L1 adipocytes [41]. kaempferol and quercetin were shown to act as weak partial agonists in the PPAR*γ* reporter gene assay, without inducing differentiation of 3T3-L1 preadipocytes as traditional PPAR*γ* agonists. When kaempferol and quercetin were added together with the PPAR*γ* agonist rosiglitazone, the 3T3-L1 differentiation was inhibited in a dose-dependent manner. Competitive ligand-binding assay confirmed that kaempferol and quercetin competed with rosiglitazone at the same binding pocket site as PPAR*γ*. These compounds were also shown with significant inhibitory effects on NO production in response to lipopolysaccharide treatment in macrophage cells in which the PPAR*γ* was overexpressed. These findings suggest that kaempferol and quercetin may act on multiple targets to ameliorate hyperglycemia [41].

Ivorra et al. studied the effects of daucosterol (*β*-sitosterol 3-*β*-glucoside) and its aglycone (*β*-sitosterol) on plasma insulin and glucose levels in normo- and hyperglycemic rats and found that oral administration of daucosterol or *β*-sitosterol increased the fasting plasma insulin levels. In addition, both compounds improved the oral glucose tolerance and increased glucose-induced insulin secretion [42]. In addition, rutin, one of the constituents of* E. alatus*, has been shown to decrease the plasma levels of glucose and lipids and increase the expression of PPAR*γ* mRNA and protein in skeletal muscles of db/db mice [43]. Rutin was also demonstrated with an AR inhibition activity (IC_50_ 3.01 *μ*M) [44]. Quercitrin, a flavonoid glycoside in* E. alatus*, was demonstrated as a noncompetitive AR inhibitor. It blocked polyol accumulation in intact rat lenses incubated in medium containing high concentration of sugars [45]. Other compounds, such as linarin (acacetin-7-O-*β*-D-rutinoside), have also been demonstrated with inhibitory activity against *α*-glucosidase [46].

### 3.1. Clinical Evidence

There have been limited clinical studies, mostly case reports, on the antidiabetic actions of* E. alatus *containing formulae (Table 8). In addition, a controlled trial, involving two groups of patients (40 patients in each group) with impaired glucose tolerance, showed that the group treated with diet and exercise intervention plus* E. alatus *formula for 1 month had significantly reduced blood glucose levels, compared to that in the diet and exercise control group. The effective rate was 80% in the* E. alatus *treatment group, compared to that of 55% in the control group [47]. It should be pointed out in most of these studies that* E. alatus *was not used lone, but in combination with other herbs; thus it is not clear if the observed effects are due to* E. alatus *or through interactions with other herbs. Thus, there may be potential bias in these findings. The current evidence for the clinical efficacy for treating diabetes is still weak. Nevertheless, these findings warrant further studies.

## 4. Conclusion

There is an increasing interest in* E. alatus* as a potential antidiabetic agent. More than 100 chemical constituents have been isolated and identified from* E. alatus*. The main chemical classes include flavonoids, terpenoids, steroids, phenylpropanoids, cardenolides, phenolic acids, and alkaloids.* E. alatus* has been demonstrated with hyperglycemic activity* in vivo*. The hypoglycemic activity* E. alatus* may be related to its effects on insulin signaling and glucose metabolism, including stimulating insulin secretion, improving affinity of insulin and receptor, increasing insulin sensitivity and tolerance, and reducing insulin resistance. It may also act as PPAR*γ* agonist and aldose reductase inhibitor. Further study on the bioactive compounds of* E. alatus* and its pharmacology may help to develop new agents for treating diabetes and diabetic complications.

## Figures and Tables

**Table 1:** Quercetin and glycosides in *E. alatus*.

Skeleton	Number	Name	R_1_	R_2_	Reference
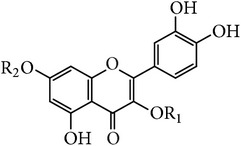	1	Quercetin	H	H	[24, 39, 48–50]
	2	Quercitrin	*α*-L-Rhamnose	H	[17, 49]
	3	Quercetin-7-O-*α*-L-rhamnoside	H	*α*-L-Rhamnose	[50]
	4	Quercetin-3,7-O-*α*-L-dirhamnoside	*α*-L-Rhamnose	*α*-L-Rhamnose	[17, 50]
	5	Quercetin 3-D-galactoside (hyperin)	3-D-Galactose	H	[17, 20]
	6	Rutin	*α*-L-Rhamnopyranosyl-(1 → 6)-*β*-D-glucopyranose	H	[39]

**Table 2 tab2:** Kaempferol and the glycosides in *E. alatus*.

Skeleton	Number	Name	R_1_	R_2_	Reference
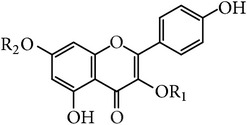	7	Kaempferol	H	H	[18, 21, 48, 49]
	8	Kaempferol-7-O-*α*-L-rhamnoside	H	*α*-L-Rhamnose	[50]
	9	Kaempferol-3,7-O-*α*-L-rhamnoside (kaempferitrin)	*α*-L-Rhamnose	*α*-L-Rhamnose	[17, 50]
	10	Kaempferol-7-O-*β*-D-glucoside	H	*α*-L-Glucose	[50]
	11	Apigenin-3-O-L-rhamnopyranoside	*α*-L-Rhamnose	H	[49]

**Table 3 tab3:** Apigenin and glycoside in *E. alatus*.

Skeleton	Number	Name	R_1_	R_2_	Reference
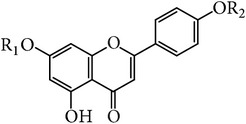	12	Apigenin	H	H	[48]
	13	Acacetin-7-O-rutinoside (Linarin)	Rutinose	H	[18]

**Table 4:** Flavanone and the glycoside in *E. alatus*.

Skeleton	Number	Name	R_1_	R_2_	R_3_	R_4_	Reference
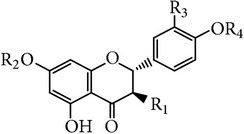	14	Dihydroquercetin	OH	H	OH	H	[49, 50]
	15	Aromadendrene	OH	H	H	H	[19, 39, 49, 50]
	16	Naringenin	H	H	H	H	[48–50]
	17	Naringin	H	Neohesperidose	H	H	[18]
	18	Hesperidin	H	Rutinose	OH	CH_3_	[50]

**Table 5:** Steroids in *E. alatus*.

Skeleton	Number	Name	R	References
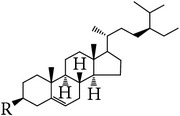	28	*β*-Sitosterol	OH	[18, 20, 24, 39, 51, 52]
	29	*β*-Sitosterone	=O	[51]
	30	Daucosterol	Glucose	[21, 48]

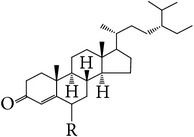	31	Stigmast-4-en-3-one (sitostenone)	H	[51, 52]
	32	6*β*-Hydroxy-stigmast-4-en-3-one	OH	[51, 52]
	33	Stigmast-4-en-3,6-dione	=O	[51]

**Table 6:** Lupane type and friedelane type triterpenes in *E. alatus*.

Lupane type	Number	Name	R_1_	R_2_	R_3_	R_4_	R_5_	References
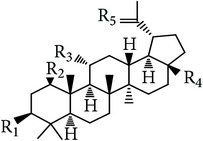	36	Lupeol	OH	H	H	CH_3_	CH_2_	[21, 48]
	37	Lupenone	=O	H	H	CH_3_	CH_2_	[24]
	38	Betulin	OH	H	H	CH_2_OH	CH_2_	[24]
	39	Betulone	=O	H	H	CH_2_OH	CH_2_	[26]
	40	Betulinic acid	OH	H	H	COOH	CH_2_	[23]
	41	Messagenin	OH	H	H	CH_2_OH	O	[26]
	42	(−)-Nepetidone	OH	OH	OH	CH_3_	O	[48]

Friedelane type	Number	Name	R_1_			R_2_		Reference

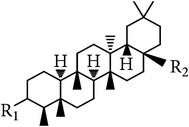	43	Epifriedelanol	OH			CH_3_		[18, 24, 39, 52]
	44	Friedelin	=O			CH_3_		[20, 24]

**Table 7:** Illustrated examples of other constituents in *E. alatus*.

Number	Name	Chemical structure	Reference
98	Usnic acid	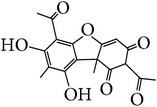	[24, 48]

99	Protocatechuic acid	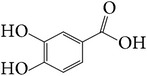	[18, 39]

100	2-Hydroxy-4-methoxy-3,6- dimethylbenzoic acid	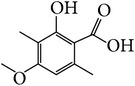	[48]

101	Benzoic acid	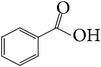	[24, 48]

102	Methyl 2,4-dihydroxy-6-methyl benzoate	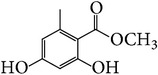	[52]

103	2,4-Dihydroxy-3,6-dimethylbenzoate	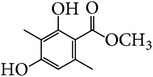	[21, 52]

104	7-Methoxy-4-methyl phthalide	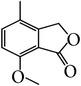	[52]

105	Caffeine	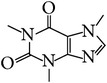	[25]

106	Caffeic acid	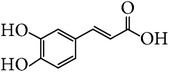	[49, 53]

107	Chlorogenic acid	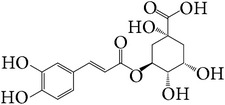	[54]

108	Vanillin	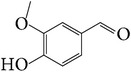	[52]

109	5-Hydroxymethyl furfural	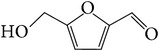	[25]

110	Dulcitol	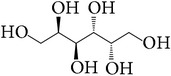	[20]

111	Grasshopper ketone	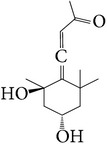	[49]

112	Suberone	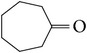	[18]

113	Syringin	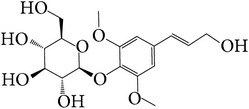	[49]

**Table 8:** Clinical studies of *E. alatus for *diabetic conditions.

Number of patients	Preparation/compound	Treatment	Outcome measures/outcome	Reference
57	*E. alatus* formula containing other herbs	Oral, daily per dose, for 3 months	Fasting blood glucose and 24 h urine glucose levels 30 cases with marked improvement 19 cases improved 8 cases no effect Total effective rate: 86%	[55]

58	*E. alatus* formula containing other herbs	Oral, daily per dose, average medication for 37.2 days.	Fasting blood glucose level 21 cases remarkable effect 28 cases effective 9 cases no effect Effective rate: 84.4%	[56]

100	*E. alatus* formula containing other herbs	Oral, daily per dose, for 4 months	Fasting blood glucose and urine glucose levels, clinical symptoms 40 cases showed remarkable effect 51 cases effective 9 cases failed Total effective rate: 91%	[57].

1	*E. alatus *decoction	Oral, daily per dose, for 20 days	Hypoglycemic effects Reduced blood and urine glucose and increased body weight	[58]

80	*E. alatus* formula containing other herbs	Oral, daily per dose, for 30 days	Fasting blood glucose, 2 h postprandial blood glucose values Treatment group: 32 cases effective (80%) and 8 cases no effect (20%) Control group: 22 cases effective (55%) and 18 cases no effect (45%)	[47]

## Abbreviations

AR: Aldose reductase
DPP-IV: Dipeptidyl peptidase IV
*E. alatus*: * Euonymus alatus*
GC-MS: Gas chromatography-mass spectrometry
LDL: Low-density lipoprotein
NO: Nitric oxide
PPAR*γ*: Peroxisome proliferator activated receptor gamma
PTP1B: Protein tyrosine phosphatase 1B
SOD: Superoxide dismutase
STZ: Streptozotocin
TNF-*α*: Tumor necrosis factor-alpha.
